# Affinities of the fern genus *Ptisana* (Marattiaceae) in the Solomon Islands, with descriptions of two new species

**DOI:** 10.3897/phytokeys.170.59471

**Published:** 2020-12-10

**Authors:** Andrew G. Murdock, Cheng-Wei Chen, Yao-Moan Huang, David Glenny

**Affiliations:** 1 Innovative Genomics Institute, University of California, Berkeley, 2151 Berkeley Way, Berkeley, California 94720, USA; 2 University and Jepson Herbaria, University of California, Berkeley, 1001 Valley Life Sciences Building, Berkeley, California 94720, USA; 3 No. 37, Lane 656, Chung Cheng Rd., Keelung City, 20246, Taiwan; 4 Taiwan Forestry Research Institute, No. 53, Nanhai Rd, Zhongzheng District, Taipei City, 10066, Taiwan; 5 Allan Herbarium, Manaaki Whenua, PO Box 40, Lincoln 7640, New Zealand

**Keywords:** Ferns, Marattiaceae, pteridophytes, *
Ptisana
*, Solomon Islands, taxonomy

## Abstract

In the process of undertaking a comprehensive review of the pteridophytes of the Solomon Islands, multiple unidentified specimens of the fern genus *Ptisana* Murdock (Marattiaceae) were collected. Morphological and molecular phylogenetic analyses as well as field observations were required to identify the Solomon Islands taxa. Four species and one variety are recognized from the Solomon Islands: *Ptisana ambulans* Murdock & C.W. Chen, **sp. nov.**, *Ptisana decipiens* Murdock & C.W. Chen, **sp. nov.**, Ptisana decipiens var. delicata Murdock & C.W. Chen, **var. nov.**, *Ptisana papuana* (Alderw.) Murdock & C.W. Chen, **comb. nov.**, and *Ptisana smithii* (Mett. ex Kuhn) Murdock. The complexities in the identification of Solomon Islands collections show the limits of morphology in the genus and illuminate a path forward for untangling the *Ptisana* taxonomy on a broader scale.

## Introduction

The country of the Solomon Islands comprises two archipelagos and nearly 1000 islands, lying to the east of Papua New Guinea and stretching across 1300 km of the Pacific Ocean to within 150 km of Vanuatu in the southeastern reaches of the country (Coleman 1966; Neall and Trewick 2008). In the course of completing a comprehensive pteridophyte flora of the Solomon Islands (Chen et al. in prep), new herbarium collections were made that included multiple unidentified members of the genus *Ptisana* Murdock, a group of large, terrestrial ferns in the Marattiaceae family, with an unsettled taxonomy in the region.

Historically, *Ptisana* was treated as part of the genus *Marattia* Sw. with a pantropical distribution. Following molecular and morphological phylogenetic analyses that found *Marattia* to be paraphyletic (Murdock 2008a), *Marattia* was split into three genera: *Ptisana*, comprising the paleotropical species, *Eupodium* J.Sm., a genus of 3–4 species in the American tropics, and *Marattia* s.s., six species restricted to the American tropics and Hawaii (Murdock 2008b). Later studies have also supported the monophyly of these genera (Senterre et al. 2014; Rothwell et al. 2018; Liu et al. 2019; Lehtonen et al. 2020). Morphologically, *Ptisana* is characterized by deeply cut, fully fused, sessile synangia, sporangial apertures that lack labia, and the presence of sutures at the attachment point of ultimate segments (Murdock 2008b).

Murdock (2008b) recognized 20 species and three varieties in *Ptisana*, placing many of the over 70 named species of Old World *Marattia* in synonymy. This was done with the caveat that some *Ptisana* species were likely overly broad as recognized, but that further work was needed to clarify some of the more challenging complexes where morphology was inconclusive. Since that time, three new species have been named in *Ptisana*, and nine new combinations have been made from earlier names in *Marattia* (Yonekura 2011; Christenhusz et al. 2011; Senterre et al. 2014; Christenhusz et al. 2018).

The prevailing challenges for taxonomists in *Ptisana* (and other marattioid genera, notably *Angiopteris*), are their size, resulting in poor, incomplete collections, and their phenotypic plasticity. Characters that are potentially taxonomically informative, e.g. ornamentation and indument of stipe bases or stipule morphology (Holttum 1978), are typically not preserved, while the easier-to-collect pinnules have characters that are often both highly labile and confusingly similar from species to species. Distinctions between many of the described species, often based on fragmentary herbarium specimens with limited comparison to other species, have long been unclear. This is especially true in the Papua New Guinea region, where a proliferation of poorly distinguished forms can be found, and no comprehensive diagnostic keys have been published. Papua New Guinea is home to the *Ptisana* with the largest segments, *Ptisana obesa* (Christ) Murdock, as well as the smallest, *Ptisana werneri* (Rosenst.) Christenh., with an ultimate segment scarcely larger than the single synangium that it bears on its short midrib. A thorough examination of herbarium specimens by Murdock (2008b) located many intermediate forms between described species in New Guinea. The wide range of morphologies with incompletely sorted characters might indicate a recent radiation in the region and warrants further collection and study.

Compared to Papua New Guinea, *Ptisana* in the nearby Solomon Islands has been poorly collected and studied until recently. While there are 16 species of *Marattia/Ptisana* described from Papua New Guinea, there have been zero species described from the Solomon Islands. The lack of unique *Ptisana* species in the Solomon Islands could simply reflect reality, not lack of attention: due to the proximity of the western islands to Papua New Guinea and habitat similarity, relatively few endemic pteridophytes have been found in the Solomon Islands (Glenny unpub.).

Among the pteridophytes of the Solomon Islands, the largest portion shares affinities with New Guinean and Malesian lineages, although Pacific Island taxa are also well represented, particularly in the southeast in the Santa Cruz group (Braithwaite 1975; Chen et al. 2017). Collections of *Ptisana* in the Solomon Islands, if identified beyond the genus level at all, have most commonly been identified in herbaria as *Ptisana ternatea* (de Vriese) Murdock (a 3-pinnate species described from Ternate in the Maluku islands), *Ptisana melanesica* (Kuhn) Murdock (a 3-pinnate species described from New Hanover in the Bismarck Archipelago, notable for its tiny ultimate segments), or *Marattia andaiensis* Alderw. (a 2-pinnate species described from eastern Papua New Guinea), indicating a likely affinity with Malesian and New Guinean *Ptisana* clades. Many of these identifications have been tentative or accompanied by question marks. Previous checklists (Foreman 1971; Henderson and Hancock 1988) included *Marattia* but were uncertain about the species. Glenny (unpub.) noted some clear differences between *P. ternatea* in the Maluku islands and the 3-pinnate form in the Solomon Islands, but retained the name citing the need for more evidence before adding new names to this difficult genus.

As part of a project to catalog the pteridophytes of the Solomon Islands (Chen et al., in prep.), additional collections were made from across the Solomon Islands, and further study was undertaken to determine the identity of the *Ptisana* species. Based on morphology, there were some indications that at least one species in the Solomon Islands was undescribed. Because morphology alone was insufficient, DNA sequencing was undertaken to aid identification and to clarify the taxonomy of *Ptisana* in the region.

## Methods

### Study area

**Figure 1.**
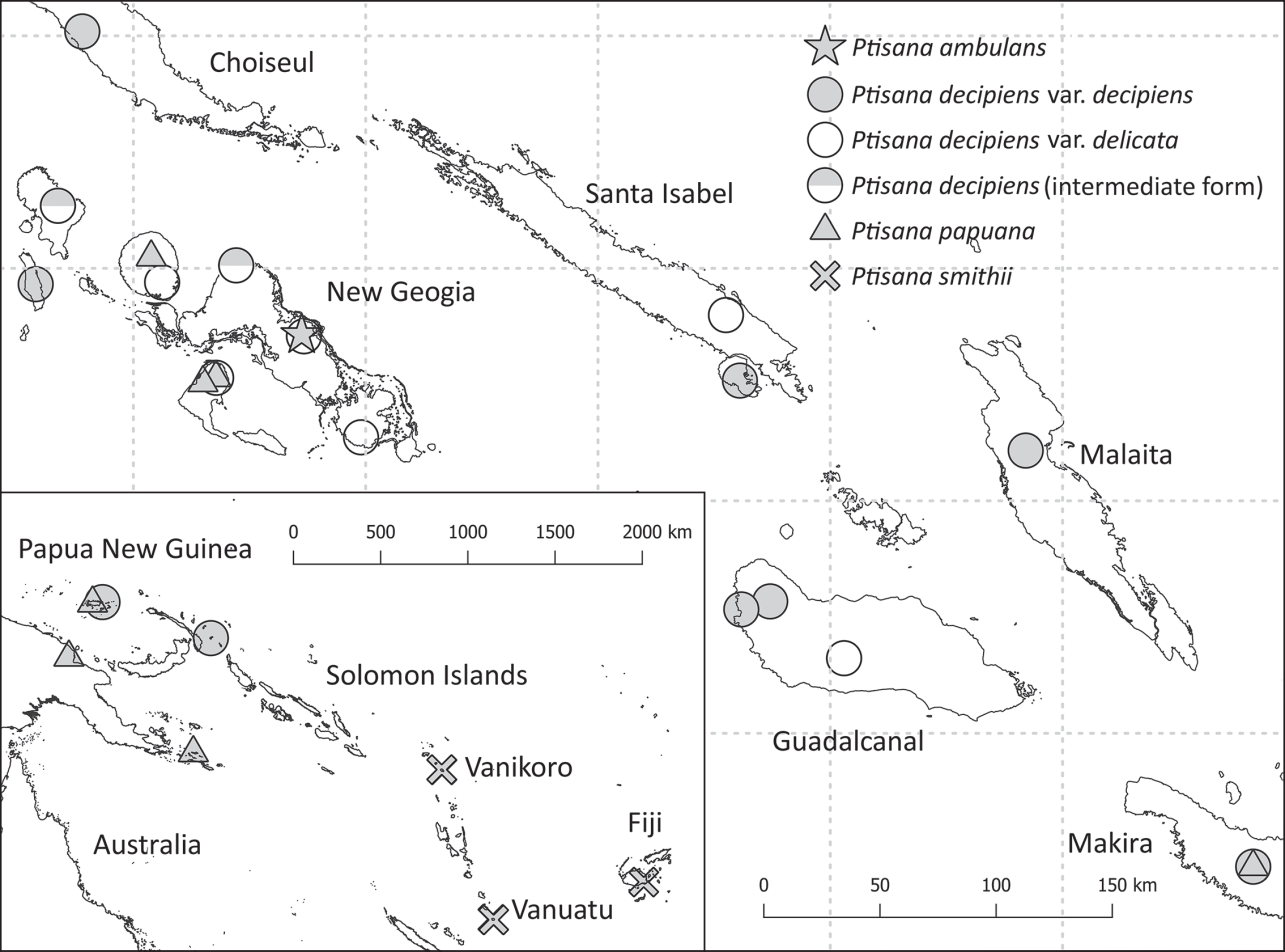
Map of the Solomon Islands showing the locations of the selected specimens examined for each taxon and collections sequenced for this paper. Specimens from Vanikoro (Santa Cruz Islands) and surrounding countries shown in inset map (bottom left).

Because the goal was to identify the *Ptisana* taxa for the Solomon Islands pteridophyte project, the study area was defined as the Solomon Islands in the political sense (Fig. 1), including the Santa Cruz Islands (Temotu Province). Biogeographically, Bougainville and neighboring Buka (Papua New Guinea) are the northernmost islands of the Solomon Islands archipelago, while the Santa Cruz Islands are the northernmost part of the Vanuatu archipelago. Notes on Bougainville and Vanuatu collections are included where relevant, but they are not included in the primary study area.

### Field observations and morphology

Due to the large size of many *Ptisana* individuals, herbarium collections frequently only capture a small portion of the characters of any plant. Field observations of characters that were difficult to preserve (e.g., stipe length and indument), as well as habitat and plant associations, filled in essential details. For taxonomic identification, all type specimens and protologues were examined for all *Marattia*/*Ptisana* species described from Papua New Guinea, Malesia, and Western Pacific regions, to compare with collections from the Solomon Islands collections. The type specimen of *P. melanesica* (Kuhn) Murdock, originally held at the herbarium of the Botanic Garden and Botanical Museum Berlin-Dahlem (B) was destroyed, but the description and accompanying illustration (Kuhn 1889) were sufficiently diagnostic.

### DNA extraction, amplification and sequencing

Fifteen samples from a range of locations and morphologies across the Solomon Islands and surrounding regions were selected for sequencing. Total DNA was extracted using a modified CTAB-Qiagen column protocol (Kuo et al. 2016). Two plastid DNA regions, *rps4* plus the *rps4*–*trnS GGA* intergenic spacer (*rps4–trnS*) (~900 bp), and the region spanning *trnS GCU* to *trnG UUC* (including *psaM* and *ycf12*) (*trnSGG*) (~1600–2100 bp) were amplified and sequenced using previously published primers and methods (Nadot et al. 1994; Smith and Cranfill 2002; Murdock 2008a).

The PCR amplifications were performed in 16 μl reactions containing ca. 10 ng template DNA, 1×Taq DNA Polymerase Master Mix RED solution (Ampliqon, Denmark), and 1 μl each of 10 μM primers. The PCR reactions were carried out in a GeneAmp PCR System 9700 (Applied Biosystems, Carlsbad, California, USA). Thermocycling conditions were the same for PCRs of these regions and comprised an initial denaturation of 2 minutes at 94 °C followed by a core sequence of 35 repetitions of 94 °C for 1 minute, 55 °C for 1 minute, and 72 °C for 1 minute followed by a final extension of 10 minutes at 72 °C. Resulting PCR products were sequenced using the same PCR primers with BigDyeTM terminator (Applied Biosystems, Carlsbad, California, USA). Sequences were deposited in GenBank. GenBank accession numbers and voucher information are provided in Appendix 1. Additional sequence data was retrieved from GenBank based on Murdock (2008a) and Lehtonen et al. (2020) for ingroup and outgroup taxa.

### DNA alignment and phylogenetic analyses

Sequence alignment was performed using MUSCLE (Madeira et al. 2019) and manually corrected using Mesquite 3.61 (Maddison and Maddison 2019). Phylogenetic analyses were performed using PhyML 3.0 (Lefort et al. 2017) and MrBayes 3.2.6 (Ronquist et al. 2012). For the Bayesian analysis, a GTR+I+G model selected by MrModeltest 2.3 based on the Akaike information criterion (Nylander 2004) was used, with 1000000 generations and four parallel chains sampled every 1000 generations, with a discarded burn-in fraction of 0.25. Support for branches was estimated using ML bootstrapping (100 replicates), and Bayesian posterior probability averaged over a majority-rules consensus tree (Fig. 1). Sequence data from each gene region was analyzed separately and concatenated both for substitution model fit and phylogenetic reconstruction. Because of agreement between data sets, both in topology and model selection, the final analysis presented here is based on the full concatenated data set. Outgroup taxa were selected based on previous phylogenetic analyses of marattioid ferns (Murdock 2008a; Liu et al. 2019; Lehtonen et al. 2020).

## Results

Morphological examination of Solomon Islands *Ptisana* collections found that individual plants could be readily sorted into two categories: plants that are consistently 2-pinnate, and those that are consistently 3-pinnate. While superficially quite similar, multiple clear distinctions were found between the 2-pinnate collections from Vanikoro (Santa Cruz Islands) and those from high elevations in the western islands of the Solomon Islands. These were identified as *Ptisana smithii* (Mett. ex Kuhn) Murdock and *Ptisana papuana* (Alderw.) Murdock, comb. nov., respectively. The common 3-pinnate collections proved more challenging to identify due to occasional intermediates between plants with small terminal segments and those with large segments. An additional 3-pinnate plant was collected from New Georgia with a suite of characters not observed in the more common forms. Based on comparison with type material and protologues, it became clear that the previous uses of *Ptisana ternatea* and *P. melanesica* were incorrect, and the Solomon Islands specimens could not be matched to any previously described species. It remained unclear how many distinct taxa were present. A full discussion of the morphological distinctions among the Solomon Islands taxa and their identification is included in the taxonomic treatment following this section.

In our molecular investigation, tree topology was consistent between ML and Bayesian analyses, recovering a monophyletic *Ptisana*. While the ML analysis showed finer resolution near the tips in some cases, these branches had uniformly low bootstrap support (<50%). While morphology can vary widely in *Ptisana*, particularly in New Guinea and Malesia, the plastid sequences across the genus are highly similar, even in the non-coding spacer regions used in this analysis, a result that is in line with previous studies (Soltis et al. 2002; Murdock 2008a; Senterre et al. 2014; Lehtonen et al. 2020). Short internal branches and polytomies were the result of limited variation in the selected sequence regions; the variation found, including insertions and deletions, was often phylogenetically uninformative.

**Figure 2.**
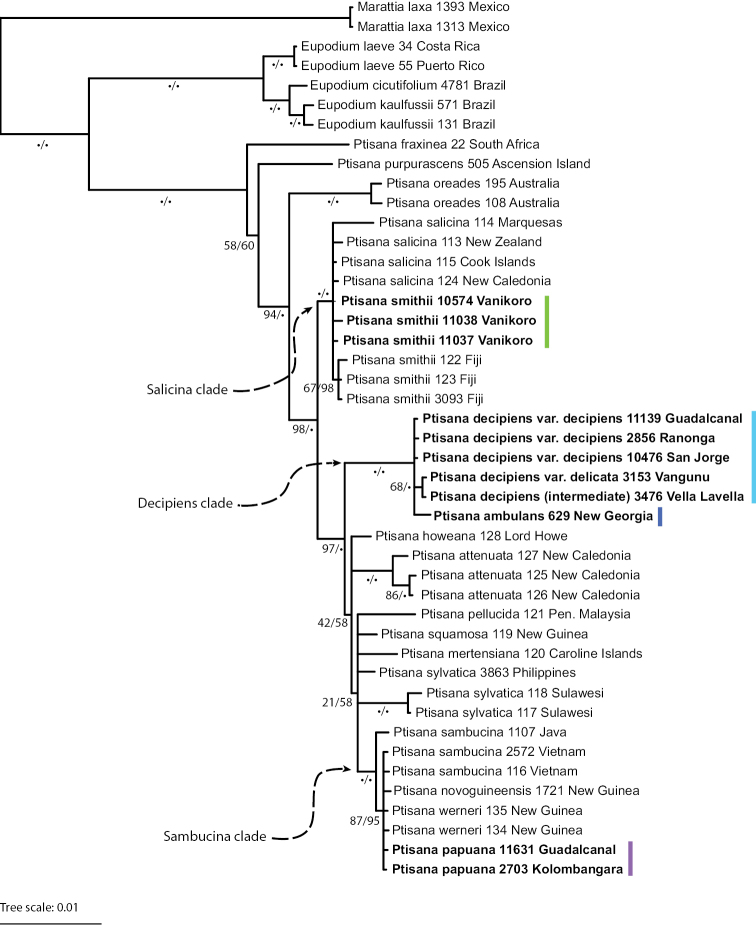
Phylogeny of *Ptisana* based on *rps4–trnS* and *trnSGG* plastid sequence data. Bayesian consensus tree, with branch support values (ML bootstrap support / Bayesian posterior probability); • = 100. The four species recognized in the Solomon Islands are marked by colored bars. Key clades discussed in text marked by arrows.

Among the plastid sequences from Solomon Islands *Ptisana*, there were five distinct haplotypes which were resolved in three different clades of the *Ptisana* phylogeny (Fig. 2). Sequences from the 2-pinnate species found in Vanikoro, identified based on morphological characters as *P. smithii*, were resolved in the Pacific island Salicina clade (highlighted in green, Fig. 2) with *P. smithii* (type from Aneityum, Vanuatu) and *P. salicina* (type from Norfolk Island). The other 2-pinnate species, identified based on morphology as *P. papuana* (highlighted in purple, Fig. 2) from the western islands, was resolved within a clade of New Guinean taxa notable for their diverse morphologies but highly similar sequences.

Sequences from the 3-pinnate taxa (highlighted in dark blue and light blue, Fig. 2) form a well-supported clade unique to the Solomon Islands, based on current sampling. Within this clade, there are three distinct haplotypes, two corresponding to the common low-elevation taxa with winged costae and no hairs subtending the synangia (including the large-segmented form often identified as *P. ternatea* and the small-segmented form often identified as *P. melanesica*), and one corresponding to a newly collected taxon from New Georgia that lacks wings on its costae and has short, uniseriate hairs subtending the synangia.

Field observations gave the first hint that the winged 3-pinnate taxa might be more similar than they first appear. David Glenny (unpub.) noted that both morphologies were found in the same habitats, never together, occasional intermediate forms were found, and the only distinction was the size of the segments. Sequences from collections with large, small, and intermediate-sized segments (highlighted in light blue, Fig. 2) were found to be identical or differ by only a single base pair over ~2700 bp. Based on the total evidence from morphological and molecular analyses, we describe the winged 3-pinnate taxa as a new species with two varieties (Ptisana decipiens var. decipiens and P. decipiens var. delicata), and the wingless taxon as a new species (*P. ambulans*) (see Taxonomic treatment section).

## Taxonomic treatment

### Terminology

The fused sporangia of *Ptisana* are referred to jointly as a synangium, the chambers of which are referred to as locules. Counts of locules per synangium refer to the entire synangium. The attachment point of the synangium is referred to as the receptacle. Axes of the leaf are referred to as the stipe (stalk below the leaf blade), rachis (main axis of leaf blade), costa (axis of a pinna), costule (axis of a pinnule on 3-pinnate plants), and midrib (axis of ultimate segment). The costule in some species is winged (readily apparent in live material, sometimes obscure in dried specimens). The swollen area at the base of each leaf division is referred to as a pulvinus. All BSIP collections are currently housed at SUVA.

**Table:** 

1	Fronds 2-pinnate	**2**
–	Fronds 3-pinnate	**3**
2	Stipes and laminae with rust-colored scales, synangia submarginal, margins strongly repand, gently serrate except at apex; Santa Cruz Islands	***P. smithii***
–	Stipes with both reddish-orange and darkened scales, synangia submedial-medial, margins lightly repand, conspicuously serrate; upland species, western islands	***P. papuana***
3	Margins entire, serrate only at apex, costulae not winged, ultimate segments ovate, synangia nearly marginal, receptacles inconspicuously hairy, uncommon (New Georgia)	***P. ambulans***
–	Margins gently serrate, costulae winged, ultimate segments oblong acuminate, varying greatly in size from location to location, synangia submarginal, receptacles glabrous, widespread	**4 (*P. decipiens*)**
4	Segments large, ultimate segments 9–18 cm long × 1.5–2.5 cm wide, 14–20 locules per synangium, fronds with 3 pairs of opposite pinnae	**P. decipiens var. decipiens**
–	Segments small, ultimate segments 2.5–5 cm long × 0.5–1 cm wide, 10–16 locules per synangium, fronds with 3–5 pairs of opposite pinnae	**P. decipiens var. delicata**

#### Ptisana ambulans

Taxon classificationPlantaeMarattialesMarattiaceae

Murdock & C.W. Chen sp. nov.

75559201-8109-5B24-B4E9-07B90D57F0AB

urn:lsid:ipni.org:names:77213329-1

Figures 3 , 6A, F

##### Type.

**Solomon Islands.** Vahole, New Georgia Island, Western Province, Solomon Islands. Under forest. 250–350 m. 28 Sep 2012. C.-W. Chen & T.-C. Hsu SITW00629. ***Holotype***: BSIP. ***Isotypes***: TAIF [421080], TNM.

##### Diagnosis.

Differs from *Ptisana decipiens* in having costae without prominent wings, nearly marginal synangia; ultimate segments ovate (versus elliptic to oblong), veins tightly spaced (ca. 0.8 mm, compared to 1.3 mm in *P. decipiens*), lamina thick, margins entire, serrated only at apex, revolute when dry, apex abruptly acuminate, uniseriate hairs subtending synangia.

##### Description.

**Figure 3.**
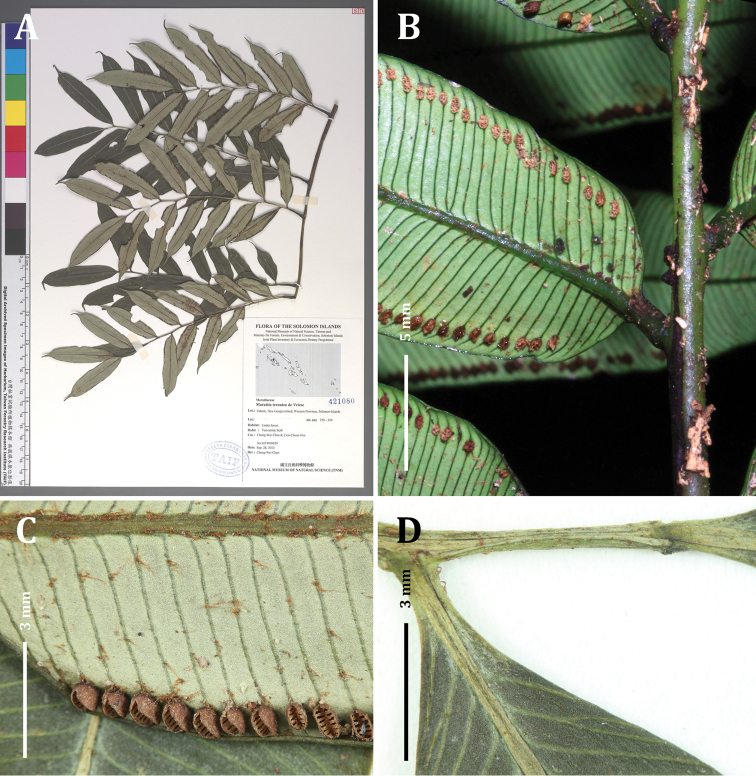
*Ptisana ambulans*: **A** type specimen **B** live plant of type collection **C** abaxial surface of ultimate segment **D** segment attachment points and terminus of the costa. Photos: C.-W. Chen.

Fronds 3-pinnate, up to 2.5 m long. Stipe circular in cross-section (stipe coloration and indument not observed). Fronds bearing 3 pairs of similarly sized pinnae on mature fronds, the terminal pair forking dichotomously at the frond apex, each pinna up to 1 m long. Swollen pulvini present at the base of all segments, green, smooth. Ultimate segments 6.5–8 cm long × 1.3–1.5 cm wide, oblong with abruptly acuminate apices (Fig, 6F); pinnule costulae slightly zigzagging and wingless (Fig. 3A, B). Laminae dark green above, pale whitish-green below, thick and coriaceous, with occasional brown-orange scales abaxially along veins and midribs (Fig. 3C, D). Veins free, ca. 0.8 mm apart, rarely dividing once near the midrib (Fig. 6A). Leaf margin entire, serrate only at apex, slightly revolute when dried. Synangia green when immature, brown after opening, one per vein, nearly marginal, set back from leaf margin by ca. 1 mm, ca. 1.5 mm long × 0.8 mm wide, 10–14 locules per synangium (Fig. 3C), receptacles bearing short, uniseriate hairs.

##### Etymology.

The epithet *ambulans* (walking) refers to the wingless costae.

##### Selected specimens examined.

Only known with certainty from one collection from New Georgia (see type above).

##### Habitat and distribution.

Low elevation forest. Altitude range: 250–350 m. Only known with certainty from one population. Solomon Islands (New Georgia).

##### Preliminary conservation assessment.

There is currently only one collection and observation of this species, but this is likely due to its similarity to the more widespread *P. decipiens*, and consequent under-collection. It is currently considered Data Deficient (DD) based on IUCN (2012).

##### Note.

In the field, this species was thought to be an atypical form of *P. decipiens*, but further examination found that both morphology and sequence data are clearly distinct, and no intermediates have been found. The presence of uniseriate hairs on the receptacle in *P. ambulans* is a character that is common in *Ptisana* but notably absent in *P. decipiens*. The rigid, thickened laminae with tightly spaced veins are reminiscent of *P. rigida* (Alderw.) Murdock, a highland species from West Papua. Together with the fact that the DNA sequences from this taxon contain unique autapomorphies, we consider this taxon sufficiently distinctive to recognize as a species. However, due to the available characters apparent on the one collection, the description here is limited and further observation is needed to supplement our understanding of this species. Examination of other collections from the Solomon Islands and Papua New Guinea have so far found no other collections of this wingless species, but we anticipate that the range likely extends beyond New Georgia.

#### Ptisana decipiens

Taxon classificationPlantaeMarattialesMarattiaceae

Murdock & C.W. Chen sp. nov.

40E1B91D-4043-5A1D-9B99-63DE96E8DCCA

urn:lsid:ipni.org:names:77213330-1

**Figure 4.**
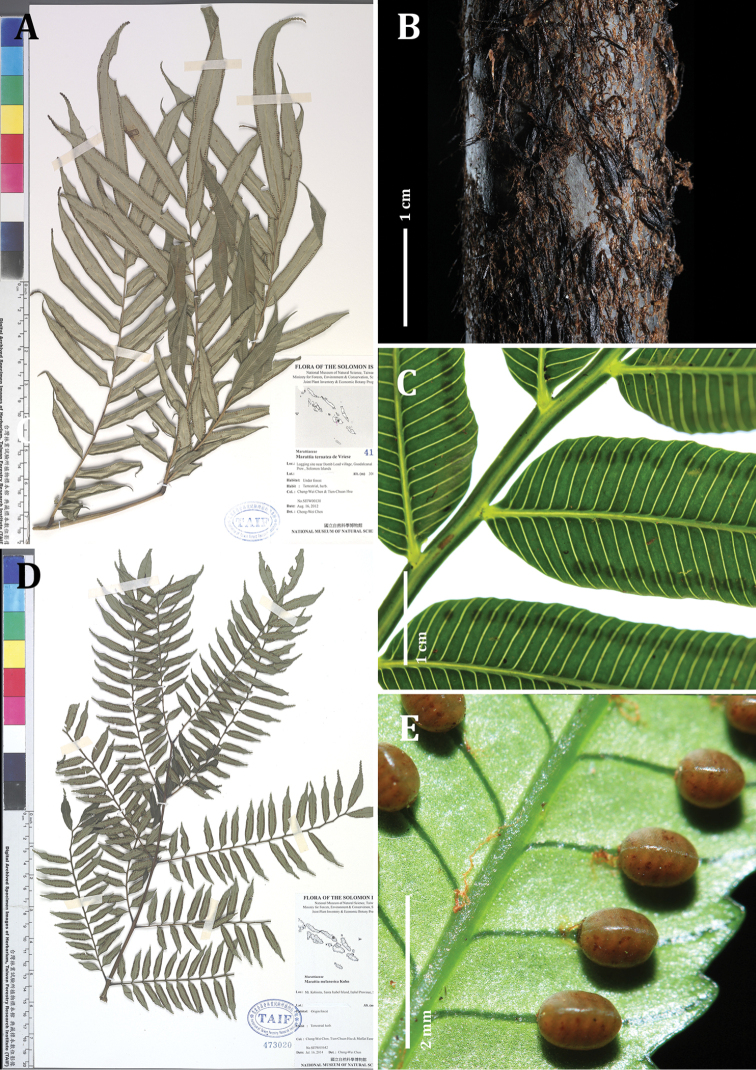
Ptisana decipiens var. decipiens: **A** type specimen, with characteristically large segments **B** stipe showing scales **C** adaxial surface of fertile segments, showing vein spacing, synangial distance from margin, and winged costa. Ptisana decipiens var. delicata: **D** type specimen, with characteristically small segments **E** abaxial surface of fertile segment with maturing synangia. Photos: C.-W. Chen.

Figures 4A–D , 6B, G

##### Type.

**Solomon Islands**. **Guadalcanal**: Logging site near Bomb Load Village, 300–400 m, 16 Aug 2012, *C.-W. Chen & T.-C Hsu SITW00130*. ***Holotype***: BSIP. ***Isotypes*** TAIF [417070, 417072], TNM.

##### Diagnosis.

Differs from *Ptisana ternatea* (de Vriese) Murdock in having glabrous receptacles, synangia that do not extend to the apex of segments, pinnules gradually reducing in size toward the base of pinnae, and pinnule apices not abruptly acuminate. Differs from *Ptisana melanesica* (Kuhn) Murdock in having larger pinnules with submarginal synangia and smaller marginal teeth. The marked variability in size of ultimate segments has not been recorded in any other *Ptisana* species.

##### Description.

Ptisana decipiens var. decipiens: Fronds 3-pinnate, up to 2.5 m long. Stipe up to 1.2 m long, round in cross-section, surface green to brown, darkening with age, with reddish-blackish scales, the broader scales being darker in color, lenticels raised (Fig. 4B). Fronds bearing 3 pairs of similarly sized pinnae on mature fronds, the terminal pair forking dichotomously at the frond apex, each pinna up to 1 m long. Swollen pulvini present at the base of all segments, green, smooth. Ultimate segments 5–12 pairs per pinnule, alternating on the costulae, largest at apex of each pinnule, smaller at the base, ultimate segments 9–18 cm long × 1.5–2.5 cm wide, elliptic to oblong with an acuminate apex; pinnule costulae gently zigzagging and clearly winged between segments (Figs 4A, 6G). Laminae herbaceous-coriaceous, dark green above, pale below, with sparse brown-orange scales along the veins and midrib abaxially. Veins free, ca. 1.3 mm apart, rarely dividing once near the midrib (Fig. 6B). Leaf margin gently serrate, more conspicuous at apex. Synangia green when immature, brown after opening, one per vein, submarginal, set back from leaf margin by 1–2 mm, ca. 1.8 mm long × 0.8 mm wide, 14–20 locules per synangium (Fig. 4C), receptacles glabrous.

##### Etymology.

The epithet *decipiens* (deceiving or misleading) refers to the morphological variation that has misled people into thinking two species were present.

##### Selected specimens examined.

**Solomon Islands. Choiseul**: Sirebe, 128 m, 4 Aug 2014, *C.-W. Chen, W.-S. Wu & M. Fanerii SITW05882* (BSIP, TAIF [474134], TNM); **Ranongga**: Qiloe, 400–700 m, 16 Aug 2013, *C.-W. Chen, T.-C. Hsu & M. Fanerii, SITW03102* (BSIP, TAIF [448596], TNM); **Guadalcanal**: Vunga Tubu, 100–500 m, 27 Jul 2014, *C.-W. Chen, T.-C. Hsu & M. Fanerii*, SITW05767 (BSIP, TAIF [472271], TNM); **Malaita**: Mt. Saranifilu, 700–800 m, 30 Jan 2015, *H.-C. Hung, C.-W. Chen & M. Fanerii SITW08836* (BSIP, TAIF[501947], TNM); **Makira**: Materato to Mt. Gasi, 910 m, 1 Jul 2015, *H.-C. Hung, C.-W. Chen & M. Fanerii SITW06724* (BSIP, TAIF [482700], TNM). **Papua New Guinea. Manus Province**: Los Negros, 17 Nov 1944, *W.H. Wagner Jr. 3277bis* (US [1860271]); **New Ireland**: Ambitle Island, 150 m, 7 Nov 2003, *W. Takeuchi 16691* (US [3481228]).

##### Habitat and distribution.

Lowland forest, most commonly in gullies, also on hillsides below ridges. Commonly in association with *Angiopteris microura* Copel. Elevation range: 0–1550 m. Common. Solomon Islands (Baga, Choiseul, Guadalcanal, Santa Isabel, Makira, Malaita, Mono, New Georgia, Nggatokae, Nggela Sule, Ranongga, Rendova, San Jorge, Ulawa, Tetepare); Bougainville; New Ireland. A collection from Fergusson Island (10 Nov 76, *J.R. Croft 68741*, BISH, K, NSW [507470], US [3324251]) may also be this species.

##### Preliminary conservation assessment.

Both P. decipiens var. decipiens and P. decipiens var. delicata are widespread in the Solomon Islands and their habitat is not currently under significant threat. This species is currently considered Least Concern (LC) based on IUCN (2012).

##### Note.

There has been inconsistent use of the infraspecific ranks “subspecies” and “variety” through time, and even different preferences between pteridologists and other taxonomists (Hamilton and Reichard 1992). We follow Yatskievych and Moran (1989), who recommend the use of subspecies in situations specifically involving geographically defined variation. Because of the overlapping ranges of the two taxa described here, and the presence of intermediate forms, which might indicate hybridization or ongoing diversification, we opted for the rank of variety in this case.

In most cases, the two varieties of *P. decipiens* are easy to distinguish based on segment size, but P. decipiens var. decipiens also has larger synangia with more locules. Occasional intermediates between the two varieties can be found, notably from Vella Lavella, New Georgia and Santa Isabel (Solomon Islands: Santa Isabel: *D. Glenny* 7211 (BSIP, W); Vella Lavella: 25 Oct 2013 *C.W. Chen, T.-C. Hsu & M. Fanerii SITW05013* (TAIF [463907], TNM); New Georgia: 13 May 2013, *Y.-H. Chang, W.-H. Wu, C.-F. Chen, C.-H. Hung & M. Fanerii SITW02317* (BSIP, TAIF [443219], TNM). The habitat of both varieties is the same, but the two varieties have not been observed together in any collection site. The range of the two varieties overlaps, but P. decipiens var. decipiens is more widespread, while P. decipiens var. delicata is more common in the Western Province.

The absence of indument on the receptacle is rare in *Ptisana*. This character was the basis for the obsolete genus *Gymnotheca* C.Presl, in which Presl included one species currently recognized in *Ptisana*, *P. mertensiana* (C.Presl) Murdock from the Caroline Islands.

#### Ptisana decipiens var. delicata

Taxon classificationPlantaeMarattialesMarattiaceae

Murdock & C.W. Chen var. nov.

853294C1-8FA5-5EA1-AAE9-E92189BD48A8

urn:lsid:ipni.org:names:77213331-1

Figures 4D, E , 6C, H

##### Type.

**Solomon Islands. Santa Isabel**: Mt. Kobinitu, 600–1000 m, 16 Jul 2014, *C.-W. Chen, T.-C. Hsu, M. Fanerii SITW05642*. ***Holotype***: BSIP. ***Isotypes***: TAIF [473020, 473021], TNM.

##### Diagnosis.

Differs from P. decipiens var. decipiens in the small size of ultimate segments, and in bearing more synangia relative to the length of the segment and synangia with fewer locules. Differs from *Ptisana melanesica* (Kuhn) Murdock in having larger pinnules with submarginal synangia, smaller marginal teeth; differs from *Ptisana kingii* (Copel.) Christenh. in having stipes without prickles or other ornamentation and having glabrous receptacles.

##### Description.

Fronds 3-pinnate, up to 2 m long. Stipe up to 1 m long, round in cross-section, surface green to brown, darkening with age, with reddish-blackish scales, the broader scales being darker in color, lenticels raised. Fronds bearing 3–5 pairs of similarly sized pinnae on mature fronds, the terminal pair forking dichotomously at the frond apex, each pinna up to 80 cm long. Swollen pulvini present at the base of all segments, green, smooth. Ultimate segments 10–15 pairs per pinnule, alternating on the costulae, largest at apex of each pinnule, smaller at the base, ultimate segments 2.5–5 cm long × 0.5–1 cm wide, elliptic to oblong with an acuminate apex; pinnule costulae gently zigzagging and clearly winged between segments (Fig. 4D, 6H). Laminae herbaceous-coriaceous, dark green above, pale below, with sparse brown-orange scales along the veins and midrib abaxially. Veins free, ca. 1 mm apart, rarely dividing once near the midrib. Leaf margin gently serrate, more conspicuous at apex (Fig. 6C). Synangia green when immature, brown after opening, one per vein, submarginal, set back from leaf margin by ca 1 mm, ca. 1.2 mm long × 0.7 mm wide, 10–16 locules per synangium (Fig. 4E), receptacles glabrous.

##### Selected specimens examined.

**Solomon Islands. Guadalcanal**: Popomanaseu, 1300–1750 m, 11 Sep 2015, *H.-C, Hung, T.-C. Hsu & M. Fanerii SITW09774* (BSIP, TAIF [515246, 515247], TNM); **New Georgia**: Vahole, 250–100 m, 25 Sep 2012, *C.-W. Chen SITW00523* (BSIP, TAIF [421034], TNM); **Vangunu**: Zaira Village to Mt. Vangunu camp site, 70–320 m, 5 Oct 2013, *C.-W. Chen, T.-C. Hsu & M. Fanerii SITW03734* (BSIP, TAIF [451625], TNM); **Rendova**: Ughele village, 700–1000 m, 26 Aug 2013, *C.-W. Chen, T.-C. Hsu & M. Fanerii SITW03381* (BSIP, TAIF [448701], TNM); **Kolombangara**: Ringgi, KFPL Nature Trail, 13 Aug 1991, *D. Glenny 3177* (BSIP [22031], W [P017081]). **Papua New Guinea. Bougainville**: Korpei, 570 m, 1 Nov 1961, *D.H. Nicolson 1531* (B, US [2415719]).

##### Etymology.

The epithet *delicata* (delicate) refers to the less robust appearance of this variety.

##### Habitat and distribution.

Solomon Islands (Choiseul, Guadalcanal, Kolombangara, Santa Isabel, New Georgia, Nggatokae, Rendova); Bougainville. Lowland forest, most commonly in gullies, also on hillsides below ridges. Commonly found in association with *Angiopteris microura* Copel. Altitude range: 0–1550 m. Common. More common in the western islands (Western Province).

##### Preliminary conservation assessment.

As with the overall species, P. decipiens var. delicata, is widespread in the Solomon Islands and its habitat is not currently under significant threat. It is currently considered Least Concern (LC) based on IUCN (2012).

#### Ptisana papuana

Taxon classificationPlantaeMarattialesMarattiaceae

(Alderw.) Murdock & C.W. Chen comb. nov.

D000A92F-FDD5-5032-A70C-5F2064AFDE76

urn:lsid:ipni.org:names:77213332-1

**Figure 5.**
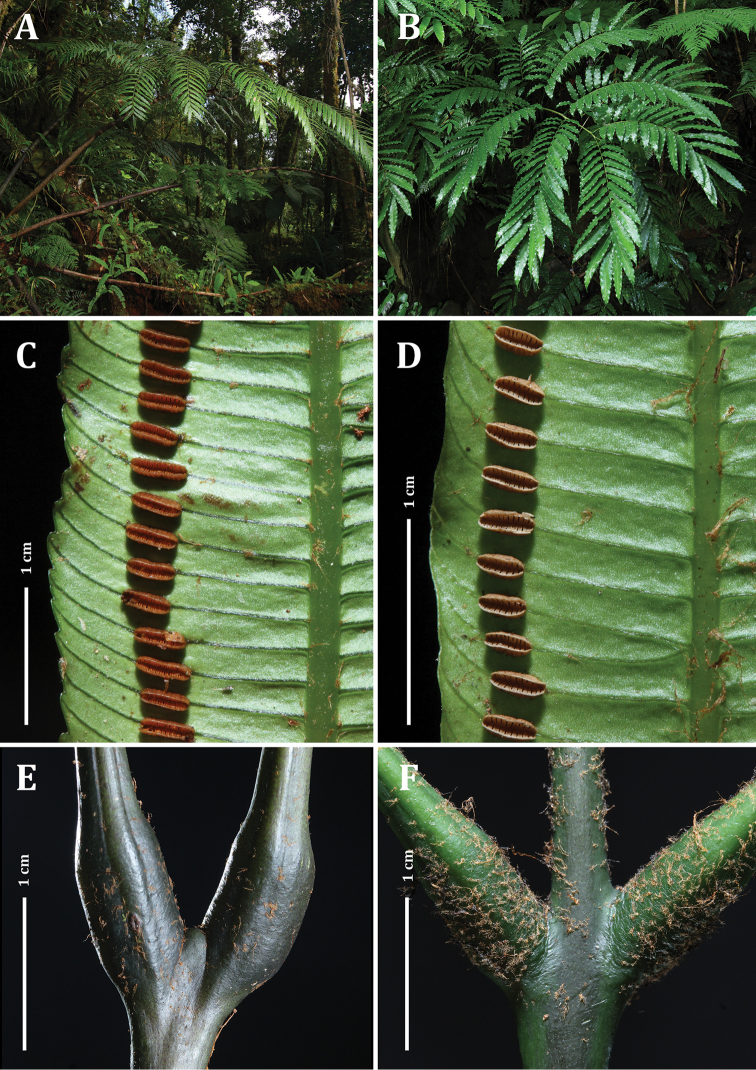
Comparison of *Ptisana papuana* (left column) and *Ptisana smithii* (right column) **A, B** whole plants *Ptisana smithii***B** showing its distinctively repand margins **C, D** fertile segments with mature synangia. *Ptisana papuana* (left) has longer synangia that reach nearly the midpoint between the margin and the midrib, and conspicuously serrate margins **E, F** Pulvini, closeup.

**Figure 6.**
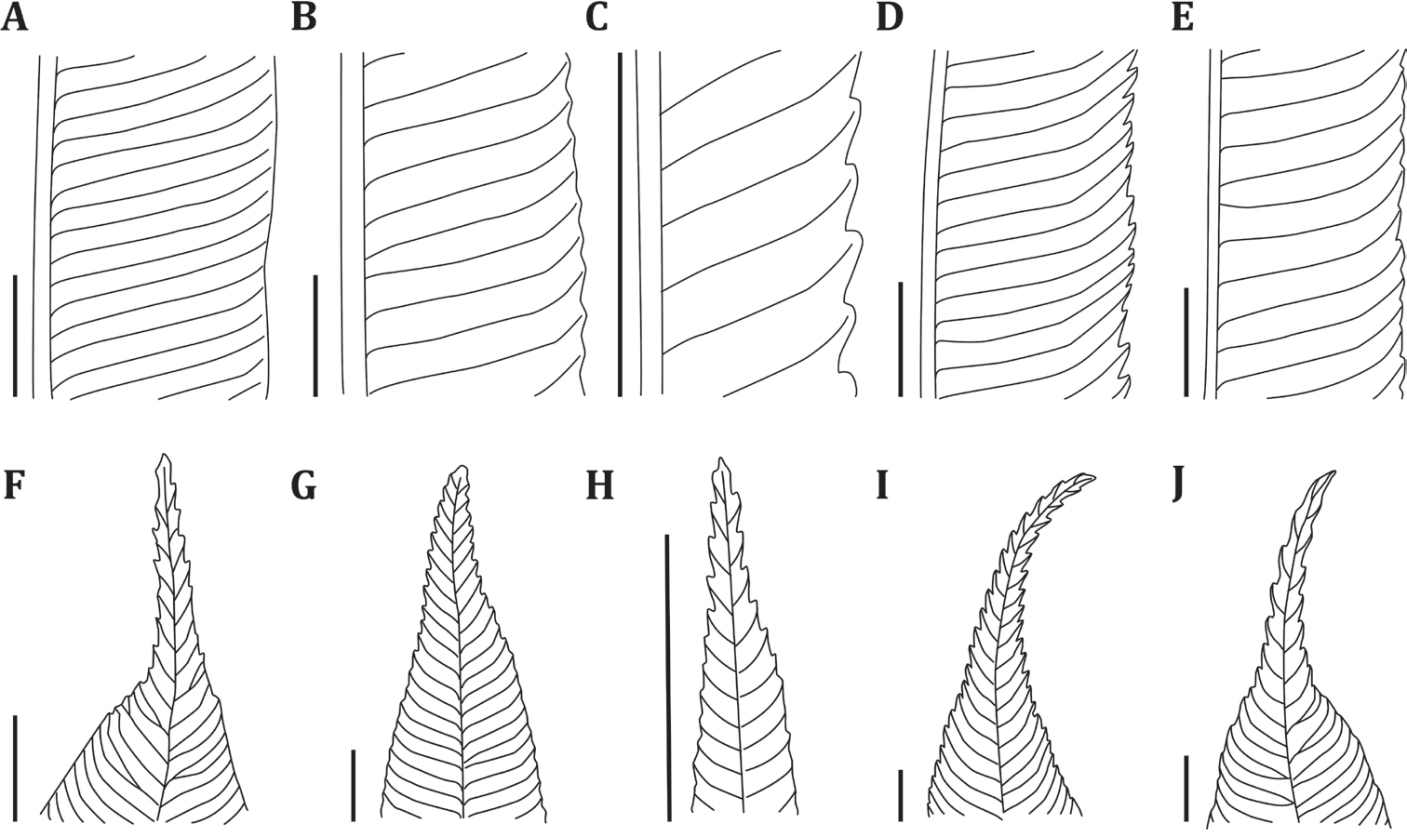
Comparison of venation and margins (**A–E**) and apices of ultimate segments (**F, G**) of the *Ptisana* species in the Solomon Islands **A, F***P. ambulans***B, G**P. decipiens var. decipiens**C, H**P. decipiens var. delicata**D, I***P. papuana***E, J***P. smithii*. Scale bars: 5 mm.

Figures 5A, C, E , 6D, I

Marattia papuana Alderw., Bull. Jard. Bot. Buitenzorg, sér. 2, 23: 17. 1916. Type: New Guinea. Constantinhafen: *Hollrung 613* (holotype: BO, photo BM!; isotype: BM!).

##### Description.

Fronds 2-pinnate, 2.4–4.0 m long. Stipe 1.5–2.0 m long, 3–6 cm diameter at the base, circular in cross-section, slightly flattened on the dorsal side, surface brown to greenish-black, densely matted with lacerate rusty orange-red scales at the base, mixed with occasional dark, undivided scales. Fronds bearing 6–8 pairs of pinnae, well-spaced on the rachis, the terminal pair forking dichotomously at the frond apex, proximal pinnae reduced in size (Fig. 5A). Swollen pulvini present at the base of all segments, dark, often with a dorsal ridge (Fig. 5E). Ultimate segments 15–18 cm long × 1.7–2.5 cm wide, narrowly oblong, base rounded but asymmetric, more cuneate acroscopically, apex acuminate (Fig. 6I). Laminae texture thick, dark green above, pale below, with occasional ragged orange scales along the veins and midrib abaxially. Veins free, ca. 1.3 mm apart, rarely dividing once near the midrib. Leaf margin strongly serrate, more pronounced at the apex, gently repand (Fig. 6D). Synangia submedial-medial, 2.0 mm long × 0.9 mm wide, 16–24 locules per synangium (Fig. 5C), receptacle bearing short hairs.

##### Selected specimens examined.

**Solomon Islands. Kolombangara**: Camp 3 to Mt. Veve, 1500–1790 m, 15 Oct 2013, *C.-W. Chen, T.-C. Hsu & M. Fanerii SITW04892* (BSIP, TAIF [465293], TNM); **Makira**: Materato to Mt. Gasi, 910 m, 1 Jul 2015, *C.-W. Chen, H.-C. Hung & M. Fanerii SITW06913* (BSIP, TAIF [482836], TNM); **Rendova**: Ughele Village, 700–1000 m, 26 Aug 2013, *C.-W. Chen, T.-C. Hsu & M. Fanerii SITW03385* (BSIP, TAIF [448705], TNM); Ughele, Rendova Peak, 11 Sep 1991, *D. Glenny 3234* (BSIP [21770], W [P017133]). **Papua New Guinea. Madang Province**: Constantinhafen, 1887, *M.U. Hollrung 613* (BM); **Manus Province**: Manus Island, Falls of Lorengau River, Nov 1945, *D.F. Grether & W.H. Wagner Jr. 4130* (UC [UC728759], US [1918547]); **Milne Bay Province**: Misima Island, Mt. Oia-Tau, 700 m, 27 Mar 1979, *J.R. Croft 71409* (US [3341352]).

##### Habitat and distribution.

Montane forest, in gullies and on hillslopes. Altitude range: 810–1550 m. Uncommon. Solomon Islands (Kolombangara, Makira, Rendova); eastern Papua New Guinea, Misima and Manus Island.

##### Preliminary conservation assessment.

*Ptisana papuana* is uncommon in the Solomon Islands, but its habitat is not currently under significant threat, and additional populations exist in Papua New Guinea. It is currently considered Least Concern (LC) based on IUCN (2012).

##### Note.

This species has been previously identified as both *Ptisana smithii* (Mett. ex Kuhn) Murdock (type from Vanuatu) and *Marattia andaiensis* Alderw. (type from eastern Papua New Guinea). Molecular analysis confirms that this is not related to *P. smithii* but is instead nested in the Sambucina clade (Fig. 2), with Malesia/New Guinea affinities. After comparison with the type specimen and the protologue of *M. andaiensis*, Solomon Islands material is a better match instead for *Marattia papuana* Alderw., described in the same publication (Van Alderwerelt Van Rosenbergh 1916). *Marattia andaiensis* is white spotted on the underside of the pinnules, has a frond that is broadest in the middle, with smaller, submarginal sori. The type collection of *Marattia papuana* was originally identified as *Marattia smithii*, a confusion echoed in the Solomon Islands.

#### Ptisana smithii

Taxon classificationPlantaeMarattialesMarattiaceae

(Mett. ex Kuhn) Murdock

3FD3A5B9-6200-5A86-810D-C522AF2210DE

Figures 5B, D, F , 6E, J

Marattia smithii Mett. ex Kuhn, Verh. K.K. Zool.-Bot. Ges. Wien 19: 584. 1869 (lectotype, designated by Murdock 2008b, pg. 748: Aneiteum, New Hebrides, Dec 1858, *Herus 5* (lectotype: P!; isolectotype: GH!).

##### Description.

Fronds 2-pinnate, up to 2.5 m long. Stipe up to 1.2 m long, 2–4 cm diameter at the base, circular in cross-section, surface dark brown to blackish-green, lighter around lenticels, with lacerate rusty scales mixed with broader brown-black scales, base of stipe bearing dense broad brown scales. Fronds bearing 5–8 pairs of pinnae, opposite to subopposite and well-spaced on the rachis, with a single terminal pinna or forking dichotomously at the frond apex, the proximal pinnae somewhat reduced in size (Fig. 5B). Swollen pulvini present at the base of all segments, pulvini of primary division often with a dorsal ridge, smooth and with a lighter color on secondary divisions (Fig. 5F). Ultimate segments 15–20 cm long × 2–2.5 cm wide, narrowly oblong, base rounded but asymmetric, more cuneate acroscopically, apex acuminate (Fig. 6J). Laminae coriaceous, dark green above, pale below, with sparse tan scales along the veins and midrib abaxially. Leaf margin lightly serrate, often strongly repand. Veins simple, ca. 1.5 mm apart, rarely dividing once near the midrib, curving toward the apex on the marginal side of each synangium (Fig. 6E). Synangia submarginal, 2.0 mm long × 0.8 mm wide, 16–20 locules per synangium (Fig. 5D), receptacles bearing short hairs.

##### Selected specimens examined.

**Solomon Islands. Vanikoro**: Rain forest, 100 m, 1928, *S.F. Kajewski 677* (F, UC [UC422670, UC1007994], MICH [1177187], US [1916159]); Ngarabu camp, 120–600 m, 17 Jun 2016, *C.-W. Chen & T.-C. Hsu & M. Fanerii SITW10574* (BSIP, TAIF [498575, 520559], TNM); Airport to Uleule River, 20–250 m, 20 Jun 2016, *C.-W. Chen & T.-C. Hsu & M. Fanerii SITW11037* (BSIP, TAIF [498870, 498871, 498872, 498873, 498874], TNM). **Vanuatu. Aneityum**: Southeast, 200 m, 26 Jul 1971, M. Schmid 3905 (L). **Fiji. Rewa Province**: Suva city, I-Suva Forest Park, 17 Sep 2013, *C.-W. Chen Wade3093* (TAIF [439749, 439750, 439751, 439752]).

##### Habitat and distribution.

Lowland forest, growing along streams and steep hillsides. Solomon Islands: Vanikoro, likely to be found on Nendo; Vanuatu; Fiji; Tonga; Samoa.

##### Preliminary conservation assessment.

*Ptisana smithii* is only known from collections from Vanikoro in the Solomon Islands, but it is widespread in adjacent island groups. It is currently considered Least Concern (LC) based on IUCN (2012).

##### Note.

The Santa Cruz group is the northern limit of the range of this species. The Salicina clade (Fig. 2), which includes *P. smithii*, is in need of revision. There are clear sequence and morphological differences from archipelago to archipelago across the Pacific. The Fijian collection sequenced for this study had synangia that were more medial than those from the Solomon Islands. Brownlie (1977) described Fijian species as having alternate pinnae, but examination of collections and photographs shows that Fijian plants have opposite or subopposite pinnae as observed in the Solomon Islands. We are retaining the use of the name *P. smithii* here because the morphology agrees so closely with collections from Vanuatu, the type locality, and we anticipate that future work will likely split *P. salicina* into a number of geographically distinct taxa.

## Discussion

The challenges of interpreting morphology in *Ptisana* are exemplified in the results of this regional study: morphology and sequence data can tell two different stories. Phenotypes that appear highly similar (e.g., *P. smithii* and *P. papuana*) can be distantly related according to sequence data, while phenotypes that appear to be quite divergent can be sequence-identical or nearly so (e.g., the small- and large-segmented forms of *P. decipiens*). In short, morphology is not sufficient for clarifying the taxonomy of the genus, and in some cases can be positively misleading. Rosenstock (1908) named the subgenus Mesocarpus after the position of the synangia proximate to the midrib in the tiny-segmented species *Ptisana werneri*. According to sequence data, *P. werneri* is scarcely different from other species with a range of synangial attachment points and both large and small segments. A combination of morphology, sequence data, and field observations was required to clarify the identities and taxonomy of Solomon Island *Ptisana*; the same will likely hold true for other regions and clades.

Based on current sampling, the Decipiens clade (Fig. 2) appears to be endemic to the Solomon Islands and nearby islands. Examination of herbarium specimens from eastern Papua New Guinea located no matching collections from the main island. While clearly distinct from *P. ternatea* from the Maluku islands, collections from near its type locality have not yet been included in molecular analyses, so it remains to be seen whether it is related to the Decipiens clade. This is a young clade that appears to be in the midst of diversification within the Solomon Islands.

The results from this study point to several groups that need additional sampling and study in the future, notably: (1) the Sambucina clade (Fig. 2), which includes the small-segmented forms from New Guinea that were lumped into *P. melanesica* by Murdock (2008b), as well as the more widespread Malesian species *P. sambucina*; (2) the Salicina clade from the South Pacific is a well-supported monophyletic group including both *P. salicina* and *P. smithii*, but it contains more than two distinct genotypes and phenotypes, and there is currently no available sequence data from either type locality; (3) *P. attenuata* from New Caledonia appears to contain some cryptic diversity and bears closer scrutiny; and (4) one of the most common Malesian species, *P. sylvatica*, requires more collection and comparative sequencing across its range.

## Supplementary Material

XML Treatment for Ptisana ambulans

XML Treatment for Ptisana decipiens

XML Treatment for Ptisana decipiens var. delicata

XML Treatment for Ptisana papuana

XML Treatment for Ptisana smithii

## Appendix 1

GenBank Accession Numbers. Taxon name, origin (ID in Fig. 2), *rps4–trnS*, *trnSGG*. Missing data –. Sequences isolated from complete plastid genomes only list one accession number. Voucher details are provided for sequences generated by this study.

*Eupodium cicutifolium* (Kaulf.) Lehtonen, Brazil (4781) (MN412590.1)

*Eupodium laeve* (Sm.) Murdock, Costa Rica (34) (EU439104.1, EU439186.1)

*Eupodium laeve* (Sm.) Murdock, Puerto Rico (55) (EU439107.1, EU439189.1)

*Eupodium kaulfussii* (J. Sm.) J. Sm. in Hook., Brazil (131) (EU439106.1, EU439188.1)

*Eupodium kaulfussii* (J. Sm.) J. Sm. in Hook., Brazil (571) (MN412589.1)

*Marattia laxa* Kunze, Mexico (1313) (MN412591.1)

*Marattia laxa* Kunze, Mexico (1393) (EU439112.1, EU439194.1)

*Ptisana ambulans* Murdock & C.W. Chen, Solomon Islands, New Georgia (629) (MW051627, MW051612), Voucher: SITW00629 (TAIF, TNM, BSIP)

*Ptisana attenuata* (Labill.) Murdock, New Caledonia (125) (EU439125.1, EU439206.1)

*Ptisana attenuata* (Labill.) Murdock, New Caledonia (126) (EU439126.1, EU439207.1)

*Ptisana attenuata* (Labill.) Murdock, New Caledonia (127) (EU439127.1, EU439208.1)

Ptisana decipiens var. decipiens Murdock & C.W. Chen, Solomon Islands, Ranongga (2856) (MW051625, MW051610), Voucher: SITW03102 (TAIF, TNM, BSIP)

Ptisana decipiens var. decipiens Murdock & C.W. Chen, Solomon Islands, San Jorge (10476) (MW051626, MW051611), Voucher: SITW10476 (TAIF, TNM, BSIP)

Ptisana decipiens var. decipiens Murdock & C.W. Chen, Solomon Islands, Guadalcanal (11139) (MW051622, MW051607), Voucher: SITW11139 (TAIF, TNM, BSIP)

*Ptisana decipiens* Murdock & C.W. Chen, Solomon Islands, Vella Lavella (3476 intermediate) (MW051624, MW051609), Voucher: SITW05013 (TAIF, TNM, BSIP)

Ptisana decipiens var. delicata Murdock & C.W. Chen, Solomon Islands, Vangunu (3153) (MW051623, MW051608), Voucher: SITW03734 (TAIF, TNM, BSIP)

*Ptisana fraxinea* (Sm.) Murdock, South Africa (22) (EU439131.1, EU439212.1)

*Ptisana howeana* (W.R.B. Oliver) Murdock, Lord Howe Island (128) (EU439128.1, EU439209.1)

*Ptisana mertensiana* (C.Presl) Murdock, Caroline Islands (120) (EU439120.1, EU439201.1)

*Ptisana novoguineensis* (Rosenst.) Murdock, New Guinea (1721) (MN412592.1)

*Ptisana oreades* (Domin) Murdock, Australia (108) (EU439129.1, EU439210.1)

*Ptisana oreades* (Domin) Murdock, Australia (195) (EU439130.1, EU439211.1)

*Ptisana papuana* (Alderw.) Murdock & C.W. Chen, Solomon Islands, Kolombangara (2703) (MW051636, MW051621), Voucher: Wade2703 (TAIF, TNM, BSIP)

*Ptisana papuana* (Alderw.) Murdock & C.W. Chen, Solomon Islands, Guadalcanal (11631) (MW051635, MW051620), Voucher: SITW11631 (TAIF, TNM, BSIP)

*Ptisana pellucida* (C.Presl) Murdock, Malaysia (121) (EU439121.1, EU439202.1)

*Ptisana purpurascens* (de Vriese) Murdock, Ascension Island (505) (EU439132.1, EU439213.1)

*Ptisana salicina* (Sm.) Murdock, New Zealand (113) (EU439113.1, EU439195.1)

*Ptisana salicina* (Sm.) Murdock, Marquesas (114) (EU439114.1, EU439196.1)

*Ptisana salicina* (Sm.) Murdock, Cook Islands (115) (EU439115.1, EU439197.1)

*Ptisana salicina* (Sm.) Murdock, New Caledonia (124) (EU439124.1, EU439205.1)

*Ptisana sambucina* (Blume) Murdock, Vietnam (116) (EU439116.1, –)

*Ptisana sambucina* (Blume) Murdock, Java (1107) (MW051634, MW051619), Voucher: Wade1107 (TAIF)

*Ptisana sambucina* (Blume) Murdock, Vietnam (2572) (MW051633, MW051618), Voucher: Wade2572 (TAIF)

*Ptisana smithii* (Mett. ex Kuhn) Murdock, Fiji (122) (EU439122.1, EU439203.1)

*Ptisana smithii* (Mett. ex Kuhn) Murdock, Fiji (123) (EU439123.1, EU439204.1)

*Ptisana smithii* (Mett. ex Kuhn) Murdock, Fiji (3093) (MW051628, MW051613), Voucher: Wade3093 (TAIF)

*Ptisana smithii* (Mett. ex Kuhn) Murdock, Solomon Islands, Vanikoro (10574) (MW051630, MW051615), Voucher: SITW10574 (TAIF, TNM, BSIP)

*Ptisana smithii* (Mett. ex Kuhn) Murdock, Solomon Islands, Vanikoro (11038) (MW051631, MW051616), Voucher: SITW11038 (TAIF, TNM, BSIP)

*Ptisana smithii* (Mett. ex Kuhn) Murdock, Solomon Islands, Vanikoro (11037) (MW051629, MW051614), Voucher: SITW11037 (TAIF, TNM, BSIP)

*Ptisana squamosa* (Christ) Murdock, New Guinea (119) (EU439119.1, EU439200.1)

*Ptisana sylvatica* (Blume) Murdock, Indonesia, Sulawesi (117) (EU439117.1, EU439198.1)

*Ptisana sylvatica* (Blume) Murdock, Indonesia, Sulawesi (118) (EU439118.1, EU439199.1)

*Ptisana sylvatica* (Blume) Murdock, Philippines (3863) (MW051632, MW051617), Voucher: Wade3836 (TAIF)

*Ptisana werneri* (Rosenst.) Christenh., New Guinea (134) (EU439135.1, –)

*Ptisana werneri* (Rosenst.) Christenh., New Guinea (135) (EU439134.1, EU439214.1)
